# Overview of mucosal immunity and respiratory infections in children: a focus on Africa

**DOI:** 10.1097/MOP.0000000000001438

**Published:** 2025-02-05

**Authors:** Richard K. Mwape, Mish-Al Barday, Marieke M. van der Zalm, Lilly M. Verhagen

**Affiliations:** a Zambart, University of Zambia, Ridgeway campus, Lusaka, Zambia; b Department of Paediatrics and Child Health, Faculty of Medicine and Health Sciences, Stellenbosch University, Cape Town, South Africa; c Department of Paediatric Infectious Diseases and Immunology, Radboud Community for Infectious Diseases, Amalia Children's Hospital, Radboud University Medical Center; d Department of Laboratory Medicine, Laboratory of Medical Immunology, Radboud University Medical Center, Nijmegen, The Netherlands

**Keywords:** sub-Saharan Africa, child, lung, pneumonia, respiratory tract infections

## Abstract

**Purpose of review:**

Given the substantial burden of respiratory tract infections (RTIs) on global paediatric health, enhancing our understanding of mucosal immunity can help us advance mucosal biomarkers for diagnosis, prognosis and possible interventions in order to improve health outcomes. This review highlights the critical role of mucosal immunity in paediatric RTIs and recent advances in mucosal interventions, which offer promising strategies to improve outcomes.

**Recent findings:**

The significant burden of paediatric RTIs and growing interest in mucosal immunity advanced our understanding of the role of the respiratory mucosal immune system in protective immunity against RTIs. Studies show that sub-Saharan Africa is disproportionately affected by paediatric RTIs with poverty-associated factors such as human immunodeficiency virus (HIV) and malnutrition as risk factors. Emerging evidence highlights the important role of the respiratory microbiome and mucosal innate and adaptive immune responses in protective immunity against RTIs.

**Summary:**

The growing interest in mucosal immunity in RTIs has not only advanced our understanding of the overall immune responses in RTIs but also created opportunities to improve RTI care through translation of knowledge from these studies into diagnostics, therapeutics, and vaccines.

## INTRODUCTION

Globally, pneumonia is the leading cause of death in children under five, accounting for 740 180 deaths and 22% of all deaths in children aged 1–5 years in 2019 [1]. Lower respiratory tract infections (LRTIs) have a variable global prevalence and disproportionally affect impoverished and young people living in low- and middle-income countries (LMICs), including sub-Saharan Africa. The highest mortality rate for pneumonia in children under 5 years of age was recorded in Somalia, with an estimated 546.8 deaths per 100 000 deaths in 2016 [2].

The causative organisms of pneumonia vary based on several factors, including geographical location, climate change, demographical differences, and level of healthcare (community or hospital-based studies). Globally, respiratory viruses are the predominant cause of childhood pneumonia with respiratory syncytial virus (RSV) and human rhinovirus (HRV) being the most commonly detected viruses in paediatric RTIs [3,4]. The PERCH study (2011–2014) investigated the causes of severe childhood pneumonia requiring hospital admission among children, aged 1–59 months, living in high disease burden, low-resource regions (Gambia, Mali, Zambia, South Africa, Kenya, Bangladesh and Thailand) in the era of routine use of vaccines against pertussis, selected pneumococcal serotypes, *Haemophilus influenzae type b* (*Hib*) and measles. The study found that viruses accounted for 61.4% of cases, bacteria for 27.3% and *Mycobacterium tuberculosis* (*Mtb*) for 5.9% among 4232 children aged 1–59 months [5^▪^]. In very severe pneumonia, viruses were less common (54.4% vs. 68%) and bacteria more common (33.7% vs. 22.8%) than in severe pneumonia. Despite widespread use of the pneumococcal conjugate vaccine (PCV), *Streptococcus pneumoniae* remained the leading cause (35.3%) of community-acquired pneumonia (CAP), probably due to serotypes not covered by the vaccine. RSV was reported as the most common cause of severe and very severe CAP in hospitalized children (31%) in the PERCH study [5^▪^]. The CHAMPS network surveillance study (2016–2022) investigated the role of pneumonia in the causal pathway of child deaths in sub-Saharan Africa and South Asia [6^▪^]. The study showed that pneumonia contributed to 40.6% of childhood deaths, with most children having more than one pathogen. The most commonly detected pathogens were *Klebsiella pneumonia,* nontypable *Haemophilus influenzae* and *Streptococcus pneumoniae*. Adenovirus (HAdV) is emerging as the third most common viral pathogen in fatal pneumonias among under-5 children in LMICs, contributing to 5.5% of all pneumonia deaths and ranking second in hospital-associated viral pneumonia deaths [7^▪^]. Various serotypes are associated with disease severity and long-term respiratory sequelae such as bronchiectasis and postinfectious bronchiolitis obliterans. Important risk factors for severe RTIs include age, nutritional status, underlying comorbidities and exposures such as smoke and household air pollution [8,9]. In LMICs, the combined impact of malnutrition, limited access to healthcare, reduced vaccination coverage and poverty-related risk factors such as human immunodeficiency virus (HIV) significantly exacerbate the burden of RTIs [10,11].

In response to the significant burden of RTIs on global paediatric health, our understanding of mucosal immunity can help us advance mucosal biomarkers for diagnosis, prognosis and possible interventions to reduce transmission and improve health outcomes. This context has stimulated a growing interest in mucosal immunity, which serves as the first line of defence by neutralizing pathogens at their entry points in the respiratory tract. Following the introduction of a nasal live-attenuated influenza vaccine in 2003, there has been an increasing focus on how mucosal immunity can contribute to respiratory health. This interest has intensified with the emergence of SARS-CoV-2, which has highlighted the need for large scale surveillance and novel noninvasive diagnostic and prognostic biomarkers for pandemic preparedness and risk stratification. In parallel, research efforts are being directed toward the development of mucosal vaccines aimed at combating pathogens during the earliest stages of infection. Novel strategies, including mucosal vector vaccines and bacteriophage-based vaccines, are being explored as part of this effort [12,13^▪^].

This review highlights the critical role of mucosal immunity in paediatric RTIs and recent advances in mucosal interventions. 

**Box 1:**
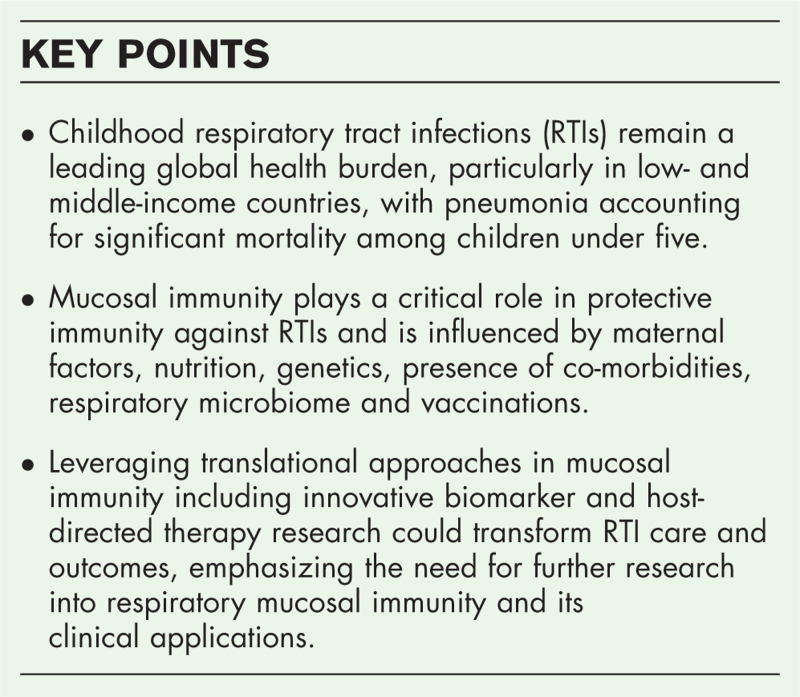
no caption available

## NATURAL HISTORY OF RESPIRATORY TRACT INFECTIONS IN CHILDREN: INNATE AND ADAPTIVE IMMUNE RESPONSES

RTIs begin with an interaction between pathogens and the host's respiratory tract, where physical and chemical barriers such as the mucociliary system and antimicrobial peptides defend against invading pathogens [14]. Pattern recognition receptors (PRRs) on respiratory epithelial cells and immune cells detect pathogen associated molecular patterns from microorganisms activating different immune responses [14,15^▪^]. Children have a higher expression of antiviral PRRs like MDA5 and RIG-I in the upper airway epithelial cells compared to adults [16].

Innate immune responses are predominantly mediated by neutrophils, natural killer cells, monocytes, macrophages, and dendritic cells [14,15^▪^]. While innate responses are typically described as nonspecific, distinct pathways are activated upon pathogen encounter. For instance, single stranded RNA viruses such as HRV are detected by the PRRs TLR7 and MDA-5 on respiratory epithelial cells, which leads to the production of cytokines such as interleukin (IL)-6 and interferon (IFN)-β that play crucial roles in antiviral defence [17]. In contrast, adaptive immune responses are classically characterized as highly specific, delayed in onset, and result in the generation of immunologic memory. Mucosa associated lymphoid tissue (MALT) structures, such as Waldeyer's ring, host adaptive immune cells including T cells and B cells [14,18]. Numerous studies have demonstrated that mucosal immunoglobulin A (IgA) is a correlate of protection against RTIs [19,20,21^▪^]. Secretory IgA plays a critical role in the fight against RTIs, although IgG, IgA and IgM all contribute to mucosal antibody responses [14,22]. Emerging evidence on mucosal polyreactive IgA challenges the traditional view of the adaptive immune system as exclusively highly specific. Unlike cross-reactive antibodies that target structurally similar antigens, polyreactive antibodies can potentially bind to several different unrelated antigens without prior exposure. This unique capability and the finding that higher levels of polyreactive IgA were associated with reduced severe RTIs in children with recurrent RTIs suggest that polyreactive IgA may function as a first-line defence, bridging innate and adaptive immunity in RTIs [21^▪^]. Further research is needed to explore the clinical applications of mucosal polyreactive antibodies.

Previous exposure to respiratory microorganisms can provide cross-protection against other pathogens. Recent studies show that HRV infection can protect against subsequent SARS-CoV-2 infections by boosting mucosal interferon stimulated genes that inhibit viral infection at different stages of the viral replication cycle [23–26].

## FACTORS AFFECTING MUCOSAL IMMUNITY

**FIGURE 1:**
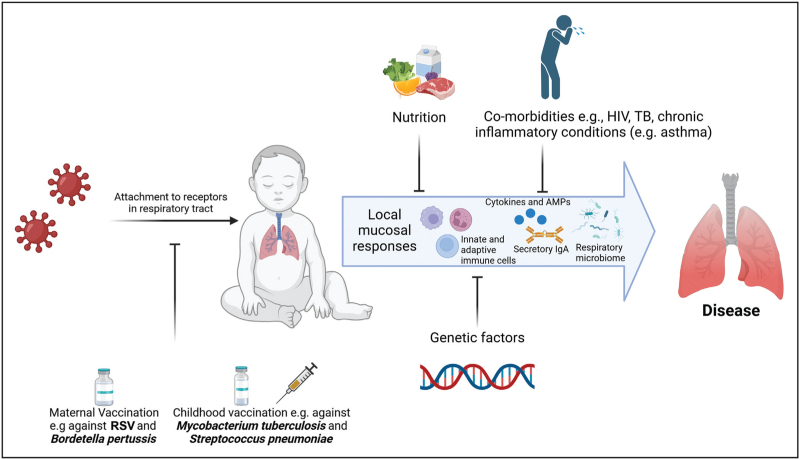
Factors that influence mucosal immune responses to paediatric respiratory tract infections. AMP, antimicrobial peptides; IgA, immunoglobulin A; RSV, respiratory syncytial virus. Created with BioRender.com.

The respiratory mucosal immune response in a young child is influenced by nutrition, genetics, presence of comorbidities, respiratory microbiome composition and vaccinations (Fig. 1). Studies in mice have shown that prenatal exposure to the bacterium *Lactobacillus johnsonii* in the maternal microbiome confers a protective phenotype against RSV in the offspring [27]. In children, the composition of the respiratory microbiome plays a critical role in the development of RTIs [28^▪^,^29^,^30^^▪▪^]. For instance, a *Corynebacterium/Dolosigranulum* respiratory microbiota profile was negatively associated with RTIs in South African and Botswanan children [29,30^▪▪^]. The respiratory microbiota of children is also influenced by maternal factors such as maternal health status, including HIV infection, breastfeeding, smoking and maternal vaccination. Exclusive breastfeeding during early life shapes the upper respiratory tract microbiome in children and protects against common RTIs [31].

## VACCINATION AND RESPIRATORY MUCOSAL IMMUNITY

Most vaccines against childhood RTIs are administered systemically and only trigger a limited immune response at the mucosal level, unlike natural infections, which begin with attachment of the pathogen to mucosal receptors. For instance, children with previous SARS-CoV-2 infection have higher spike-specific salivary IgA2 than vaccinated children [32^▪▪^]. Systemic vaccination against viral RTIs predominantly induces mucosal IgA1, which is mainly involved in immune homeostasis, whereas natural infection induces mucosal IgA2, which promotes inflammatory activity [32^▪▪^,^33^]. Hybrid immunity, defined as the immune protection resulting from the combination of natural infection and vaccination, is found to offer the most comprehensive protection against SARS-CoV-2 and *Bordetella pertussis* at both mucosal and systemic levels [34^▪▪^,^35^,^36^]. The mechanisms driving mucosal immunity following systemic vaccination remain incompletely understood.

Maternal vaccination also plays a vital role in infant RTI protection. Maternal vaccination against *Bordetella pertussis* and RSV has been shown to protect infants by transferring maternal antibodies via the placenta [37–39]. Maternal vaccination also modulates T-helper (Th) cell responses (Th1, Th2 and Th17) in infants following primary and booster vaccination. Notably, mucosal Th17 cells are critical for the clearance of *Bordetella pertussis*[40,41].

## NUTRITION AND RESPIRATORY MUCOSAL IMMUNITY

Dietary components can directly impact lung health or serve as an energy source for immune function, with their metabolites acting as potent immunomodulators [42]. There are a variety of alveolar macrophage surface receptors that allow them to sense the environment and send signals to stromal cells of the lung that maintain homeostasis or allow the perception of changes in the inhaled environment. These signals can be activators such as TLR2, TLR4, TLR6, IL-1R, IFNγR and TNFR, which are generally triggered by poor nutrition, infection, antibiotic use, pollution or microbiota restriction. Signals that may act as suppressors include CD200R, SIRPα, mannose receptor, TREM2, IL-10R and TGFβR, which are related to homeostasis and are induced under conditions of a balanced diet, minimal infections, limited antibiotic use and a diverse microbiota [42]. Undernutrition, encompassing stunting, wasting and/or deficiencies of essential micronutrients, significantly increases the risk of pneumonia and (pulmonary) tuberculosis (TB) in children, highlighting the link between nutrition and respiratory health [43,44]. Conversely, obesity is associated with reduced levels of secretory IgA, predisposing affected children to RTIs [45]. Mucosal production of antimicrobial peptides and IgA-mediated defences develops gradually during the first year of life, contributing to the heightened susceptibility of infants to RTIs [46]. Breastfed infants benefit from protective antimicrobial peptides, such as lysozyme and lactoferrin as well as passive transfer of secretory IgA, which strengthen their mucosal immune system [47].

## HIV EXPOSURE AND RESPIRATORY MUCOSAL IMMUNITY

According to UNICEF, approximately 685 children became infected with HIV and approximately 250 children died from AIDS related causes every day in 2023 [48]. Due to the immune compromised states, children living with HIV are at risk of opportunistic infections such as pneumocystis, recurrent pneumonia and TB [49]. HIV-exposed but uninfected infants are also at risk of LRTIs, especially in the first 2 years of life. This is linked to immune activation, with decreased anti-inflammatory cytokines and increased proinflammatory cytokines in cord blood, thymic dysfunction impairing T-lymphocyte response to infections and altered humoral immunity and weakened vaccine responses [49]. Maternal HIV, antiretroviral therapy use, and suboptimal maternal health further affect the infant's immune development. The impact of HIV on respiratory mucosal immunity remains insufficiently understood, but the mechanisms of systemic immune dysregulation observed in HIV-exposed and infected children suggest potential vulnerabilities. Declining CD4^+^ T cell count, and immune activation driven by persistent antigenic stimulation may impair the recruitment and function of respiratory mucosal immune cells such as macrophages and dendritic cells [50]. Additionally, abnormalities in T-lymphocyte function, particularly in Th17 cells, may exacerbate susceptibility to RTIs. Altered cytokine profiles, including increased pro-inflammatory cytokines and reduced anti-inflammatory cytokines, could further disrupt mucosal homeostasis, weakening the ability to mount an effective localized response [50]. These dysfunctions may not only increase the incidence of RTIs but also reduce the efficacy of mucosal vaccines in HIV-infected and HIV-exposed, uninfected children.

## MUCOSAL IMMUNITY AND TRANSLATIONAL RESEARCH TO IMPROVE RESPIRATORY TRACT INFECTION CARE

**FIGURE 2:**
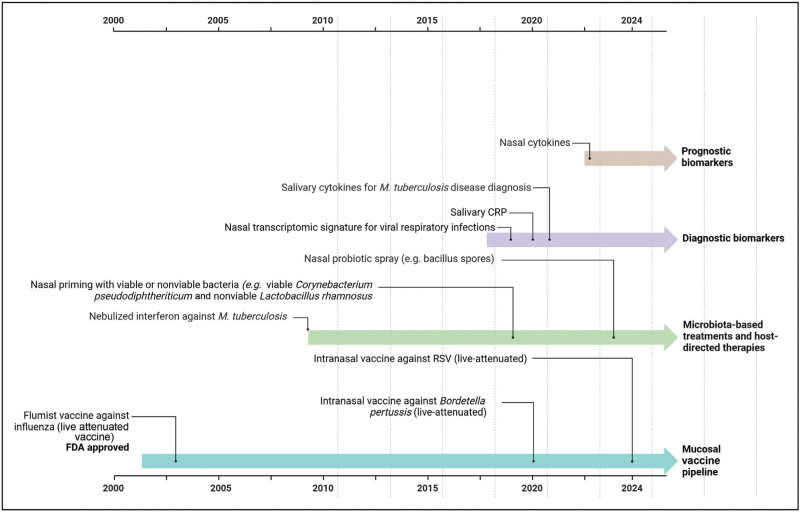
Overview of translational advances in mucosal immunity to improve respiratory tract infection care. Created with BioRender.com.

The robust protective effects observed following natural infections with respiratory pathogens underscore the potential of mucosal immune responses as an attractive target for diagnostic approaches, novel vaccine development and therapeutic interventions to improve RTI care (Fig. 2).

## MUCOSAL BIOMARKERS FOR DIAGNOSTIC AND PROGNOSTIC APPLICATIONS IN RESPIRATORY TRACT INFECTIONS

A variety of blood serum biomarkers have been explored for use as diagnostic or prognostic markers for acute RTIs, but to date, none can reliably discriminate between viral and bacterial RTIs or predict disease progression [51–53]. Mucosal immune response products can easily be measured in noninvasive samples such as saliva. While mucosal samples have traditionally been used for pathogen detection, recent research has shifted towards host response mucosal markers for RTI diagnosis and treatment monitoring.

Unique mucosal immune signatures have been described for RSV, which show potential for use as biomarkers in monitoring treatment response or diagnostics [54,55]. Salivary C-reactive protein (CRP) has emerged as an alternative to serum CRP as a diagnostic marker of pneumonia and has been shown to rise during pneumonia and fall following infection resolution [56,57]. A study in Egypt found that salivary CRP in combination with mean platelet volume was a useful marker in diagnosis of late-onset neonatal pneumonia [56]. Recently, nasal cytokines MCP-3 and CCL23 were found to be higher in children with severe pneumonia, and require further investigation as prognostic biomarkers [58^▪^,^59^].

The diagnosis of paediatric TB remains a challenge due to the paucibacillary nature of the disease in children. The WHO has prioritized the development of a nonsputum based diagnostic tool, which has subsequently fuelled biomarker research in saliva and nasopharyngeal samples. Although differences have been detected in saliva markers compared to serum markers, none of the markers showed significant differences between active TB and other respiratory diseases [60]. Some salivary host markers such as IL-1β, IL-23, ECM-1, HCC1, fractalkine, CRP and fibrinogen have shown promise in diagnosing active TB in adults [61–63]. However, results are highly variable, with only CRP and fractalkine showing consistent trends in different studies [61–64]. Most studies have been conducted in adults, while there is no recent work in children. There is a need for further evaluation of TB mucosal biomarkers in paediatric populations.

## MUCOSAL INTERVENTIONS FOR RESPIRATORY TRACT INFECTIONS

The benefits of mucosal vaccination, including robust local immune responses and minimally invasive administration, make mucosal vaccines well suited for mass immunization, particularly in low-resource settings. Mucosal vaccines minimize healthcare-associated infection risks and do not require skilled personnel for administration [65,66]. Currently, Flumist is the only FDA-approved mucosal vaccine for children targeting a respiratory pathogen. Flumist is an intranasal vaccine against influenza that induces strong mucosal IgA and systemic IgG responses in children [67,68]. In contrast, daily intranasal administration of palivizumab – a monoclonal antibody – was ineffective in preventing RSV infection in late preterm infants [69]. Recently, an intranasal live-attenuated RSV vaccine candidate was shown to be immunogenic in RSV seronegative children, however, it was also associated with rhinorrhoea in vaccinees and therefore requires further evaluation [70^▪▪^]. For bacterial causes of RTIs, the most promising advances are in the development of a mucosal pertussis vaccine. The ILiAD Biotechnologies developed BPZE1, an investigational intranasal vaccine against *Bordetella pertussis* that has been shown to induce broad secretory IgA responses in clinical trials [71,72].

Mucosal delivery of microbiota-based treatments and host-directed therapies have been investigated for use in RTIs. For example, nasal priming with viable or nonviable bacteria (e.g. *Corynebacterium pseudodiphtheriticum* or *Lactobacillus rhamnosus*) has been shown to have a protective effect against primary RSV infection and secondary pneumococcus infection in mice [73,74]. Recent studies have shown that a nasal probiotic spray reduces influenza and RSV viral loads and shortens recovery time in infected children [75,76]. These studies show that the effect of beneficial bacteria on the mucosal immune response to infections is strain-specific and offers a promising role for microbiota-based therapies as prophylaxis against RTIs, which requires further investigation in clinical trials. Mucosal host-directed therapies such as nebulized recombinant interferon-gamma1b as an adjuvant to conventional anti-TB drugs have been shown to improve symptoms and clearance of *Mtb* from the lungs in preclinical studies [77,78].

## CONCLUSION AND FUTURE PERSPECTIVES

Childhood RTIs remain a leading global health burden, particularly in LMICs, with pneumonia accounting for a significant mortality rate in children under five. Poverty-associated factors such as malnutrition and limited healthcare access exacerbate RTIs, while maternal and nutritional influences shape mucosal immunity in early life. Immunizations have reduced RTI incidence, yet challenges persist, including incomplete pathogen coverage. Advances in mucosal interventions, including vaccines, diagnostics, and microbiota-based treatments, offer promising solutions, particularly in resource-limited settings. Utilizing these approaches could transform RTI management and outcomes, emphasizing the need for further research into respiratory mucosal immunity and its clinical applications.

## Acknowledgements

*The authors would like to acknowledge all the clinicians, healthcare workers, scientists, and caregivers who dedicate their time to paediatric infectious disease research and clinical care.*

### Financial support and sponsorship

*This work is part of the VERDI project (101045989) which is funded by the European Union. Views and opinions expressed are however those of the author(s) only and do not necessarily reflect those of the European Union or the European Health and Digital Executive Agency. Neither the European Union nor the granting authority can be held responsible for them.*

*M.M.vd.Z. is supported by a career development grant from the EDCTP2 program supported by the European Union (TMA2019SFP-2836 tuberculosis lung-FACT2), the Fogarty International Centre of the National Institutes of Health (NIH) under Award Number K43TW011028, and a researcher-initiated grant from the South African Medical Research Council.*

*L.M.V. is supported by a Hypatia Fellowship funded by the Radboud University Medical Center, Nijmegen, The Netherlands, and by The Netherlands Organization for Health Research and Development (ZonMW VENI grant, grant number 09150162010077).*

### Conflicts of interest

*There are no conflicts of interest.*

## REFERENCES AND RECOMMENDED READING

Papers of particular interest, published within the annual period of review, have been highlighted as:

▪ of special interest
▪▪ of outstanding interest
